# Value of urinary lipoarabinomannan levels for tuberculosis diagnosis and monitoring of therapy

**DOI:** 10.3389/fmicb.2025.1653031

**Published:** 2025-08-20

**Authors:** Yiqun Xiong, Zhihong Shen, Bo Dong, Ying Wang, Ying Zhu, Hongxia Wei, Dongliang Zhang, Yang Che

**Affiliations:** ^1^Department of Infection, Ningbo Yinzhou No.2 Hospital, Ningbo, China; ^2^Graduate School, Lyceum of the Philippines University - Batangas, Batangas City, Philippines; ^3^Institute of Tuberculosis Prevention and Control, Ningbo Municipal Center for Disease Control and Prevention, Ningbo, China

**Keywords:** tuberculosis, lipoarabinomannan, treatment monitoring, diagnostic accuracy, predictive modeling

## Abstract

**Background:**

The urinary lipoarabinomannan (LAM) assay has emerged as a promising tool for tuberculosis (TB) diagnosis and treatment monitoring. This study aimed to evaluate the diagnostic and monitoring performance of LAM compared to Acid-fast bacilli (AFB), Mycobacteria Growth Indicator Tube (MGIT), and GeneXpert, and to establish its clinical utility in a stratified TB population.

**Methods:**

A prospective cohort study included TB patients stratified by AFB/MGIT status into three groups. Diagnostic accuracy was tested against composite reference standard (CRS). Early monitoring performance was assessed via serial LAM measurements during 12-week treatment. ROC/KM/Cox analyses determined optimal thresholds and predictors of LAM conversion.

**Results:**

Against CRS, LAM demonstrated a sensitivity of 58.75%, which was numerically higher than AFB smear (45.00%, *p* = 0.082) and comparable to MGIT culture (58.75%, *p* = 1.00), but numerically lower than GeneXpert (61.25%, *p* = 0.205). In the early monitoring phase, LAM showed sustained positivity in 11.54–51.72% at week 12, compared to <15% for other methods. The diagnostic-monitoring quadrant analysis revealed LAM’s optimal positioning for monitoring (mean conversion time 4.63–11.49 weeks), compared to 0–8.25 weeks for other methods. A combined model incorporating baseline PreLAM and week 4 change (ΔLAM) showed the highest predictive value for 12 weeks conversion (AUC = 0.871–0.943). Multivariate cox analysis identified ΔLAM as independent predictors in total cohort (HR = 0.013, *p* = 0.001) and double positive group (HR = 0.020, *p* = 0.002).

**Conclusion:**

Urinary LAM serves as a dual-role biomarker, providing moderate diagnostic sensitivity and dynamic monitoring signals reflecting early bacillary response to therapy. The PreLAM+ΔLAM model enables early treatment response assessment for personalized therapy.

## 1 Introduction

Tuberculosis (TB) remains one of the deadliest infectious diseases worldwide, with an estimated 10 million new cases and 1.5 million deaths annually (World Health Organization, 2024). Despite significant progress in TB control, critical gaps remian in both diagnosis and treatment monitoring (World Health Organization, 2024). Early and accurate diagnosis, coupled with effective monitoring of treatment response, is essential to reducing TB transmission and improving patient outcomes (Teibo et al., 2024).

Current TB diagnostic methods each face significant challenges. Acid-fast bacilli (AFB) smear microscopy, though widely available, suffers from variable and often low sensitivity (Huang et al., 2022). This limitation is particularly pronounced in paucibacillary TB, including child TB (Shingadia and Novelli, 2003), extrapulmonary TB (Bajracharya et al., 2022), or HIV coinfected TB (Bajracharya et al., 2022). Mycobacterial Culture is the gold standard for TB diagnosis, mainly including solid and liquid Culture. Mycobacteria Growth Indicator Tube (MGIT), a key liquid culture technique. Mycobacterial Culture requires specialized biosafety facilities and has a prolonged culture time (6–8 weeks), limiting its utility in routine clinical practice (Huang et al., 2022). Although GeneXpert MTB/RIF has revolutionized TB diagnostics with its rapid results, simultaneous detection of rifampicin resistance (beneficial for multidrug-resistant TB (MDR-TB) screening), its high cost and infrastructure requirements restrict accessibility in resource-limited settings (Brown et al., 2021). Crucially, all sputum-dependent methods – including smear microscopy, culture, and GeneXpert – are limited by sample quality. Results are significantly compromised for patients who cannot produce sputum, produce scant sputum, or provide poor-quality samples (Oliveira da Silva et al., 2024; Kiflie et al., 2022). Non-sputum-based alternatives, such as urine lipoarabinomannan (LAM) detection, offer potential to overcome this limitation (Kasule et al., 2024).

Beyond diagnosis, monitoring treatment response remains a major challenge. Conventional methods, such as smear microscopy and Mycobacterial Culture, often fail to provide real-time insights into treatment efficacy, especially in patients who rapidly convert to negative early in therapy (Heyckendorf et al., 2022). This creates a critical gap in identifying patients at risk of treatment failure or relapse, highlighting the need for more reliable and accessible monitoring tools.

The urinary lipoarabinomannan (LAM) assay has emerged as a promising point-of-care test for TB diagnosis and monitoring. LAM, a component of the mycobacterial cell wall, is shed into urine during active infection, offering a non-invasive and easily accessible sample (Flores et al., 2021). Early-generation LAM tests, including the Abbott Determine TB LAM Ag (AlereLAM) and Fujifilm SILVAMP TB LAM Assay (FujiLAM), exhibited suboptimal sensitivity (Broger et al., 2020; Broger et al., 2023). AlereLAM demonstrated sensitivities of 42% in HIV-positive and 18% in HIV-negative population, while FujiLAM showed 70.7 and 53.2% (Broger et al., 2020) in these groups, respectively (Broger et al., 2023). This limited performance, particularly in HIV-negative individuals, restricted their utility primarily to HIV-positive patients with advanced immunosuppression (World Health Organization, 2019). These limitations stemmed from several factors: inadequate analytical sensitivity of lateral flow immunoassays, suboptimal antibody affinity in earlier test formulations (Li et al., 2024), cross-reactivity with nontuberculous mycobacteria (NTM) species (Calhoun et al., 2024) (particularly limiting its diagnostic utility for tuberculosis in people living with HIV) (Liu et al., 2022), and host immunity (Li et al., 2025).

Recent advancements in LAM technology, particularly the development of advanced chemiluminescence methods (AIMLAM), have addressed many of these limitations. By incorporating chemiluminescence detection and optimized urine processing protocols, AIMLAM achieves significantly enhanced sensitivity, exceeding 50–55% in HIV-negative populations—a performance comparable to GeneXpert MTB/RIF in some settings (Li et al., 2025; Gao et al., 2024). This technological breakthrough has expanded the potential utility of LAM testing beyond HIV-coinfected individuals to include the broader TB patient population.

While previous studies have demonstrated the diagnostic utility of AIMLAM (Li et al., 2025; Gao et al., 2024), its role in treatment monitoring remains largely underexplored. Existing evidence suggests that LAM levels may correlate with mycobacterial load and treatment response (Jones et al., 2022), but systematic comparisons with conventional microbiological methods are lacking.

Critical gaps persist regarding AIMLAM’s performance stratified by mycobacterial load, its comparative utility against standard microbiological methods during treatment, and its role as a predictive biomarker for treatment response in HIV-negative patients—who bear most of the global TB burden yet remain underserved by LAM diagnostics s (World Health Organization, 2024).

This study aims to address these critical gaps by: 1. evaluating the diagnostic performance of AIMLAM against a composite reference standard (CRS) compared to AFB, MGIT, and GeneXpert; 2. stratifying patients based on AFB and MGIT results to assess AIMLAM’s differential utility across disease severity spectra; and 3. establishing a predictive model for early treatment response using serial LAM measurements. Through integrating diagnostic accuracy, longitudinal monitoring, and predictive analytics, this research seeks to redefine the clinical utility of LAM testing in TB management.

## 2 Materials and methods

### 2.1 Study design and population

This prospective cohort study was conducted at Ningbo Yinzhou No.2 Hospital between January 2024 and February 2025. We enrolled consecutive patients aged ≥18 years with suspected pulmonary TB who met the following inclusion criteria:

1 Presence of ≥1 clinical symptom suggestive of pulmonary tuberculosis:
 - Persistent productive cough (≥2 weeks).
 - Hemoptysis or blood-tinged sputum.
 - Documented fever (axillary temperature ≥37.3 °C).
 - Drenching night sweats.
 - Unintentional weight loss (≥5% body weight within 3 months).
2 Capable of producing adequate sputum and urine specimens.
3 Willingness to provide written informed consent.

Exclusion criteria included:

1. Prior TB treatment within the last 6 months.
2. HIV infection.
3. Extrapulmonary tuberculosis.
4. Drug-resistant tuberculosis (including mono/poly/multidrug-resistant strains).
5. Incomplete microbiological test results (AFB smear, MGIT culture, GeneXpert MTB/RIF, or AIMLAM assay).

A total of 200 suspected tuberculosis patients visited the infectious department of Ningbo Yinzhou No.2 Hospital. Seventy patients were excluded based on the following criteria:

1. Extrapulmonary TB (*n* = 20) (including 5 cases of intestinal tuberculosis, 4 cases of tuberculous peritonitis, 4 cases of renal tuberculosis, 3 cases of lymph node tuberculosis, 2 cases of skeletal tuberculosis, 1 case of ocular tuberculosis, and 1 case of epididymal tuberculosis).
2. Active TB treatment at enrollment (*n* = 2).
3. Loss to follow-up during the 3-month monitoring period (*n* = 3).
4. History of TB within the previous 6 months (*n* = 2).
5. Incomplete longitudinal AFB/MGIT/Xpert/LAM monitoring data during the first 3 months (*n* = 33).
6. Drug-resistant TB (including mono/poly/multidrug-resistant strains) (*n* = 10).

Ultimately, 130 participants were included in the final analysis: 80 pulmonary TB patients (PTB) and 50 non-TB controls. Based on AFB smear and MGIT culture results, PTB were stratified into three groups:

1. Double-positive group (DP): AFB-positive and MGIT-positive (*n* = 29).
2. Single-positive group (SP): AFB-negative and MGIT-positive or AFB-positive and MGIT-negative (*n* = 25; comprising 18 AFB-MGIT+ and 7 AFB + MGIT-).
3. Double-negative group (DN): AFB-negative and MGIT-negative (*n* = 26).

**Figure 1:**
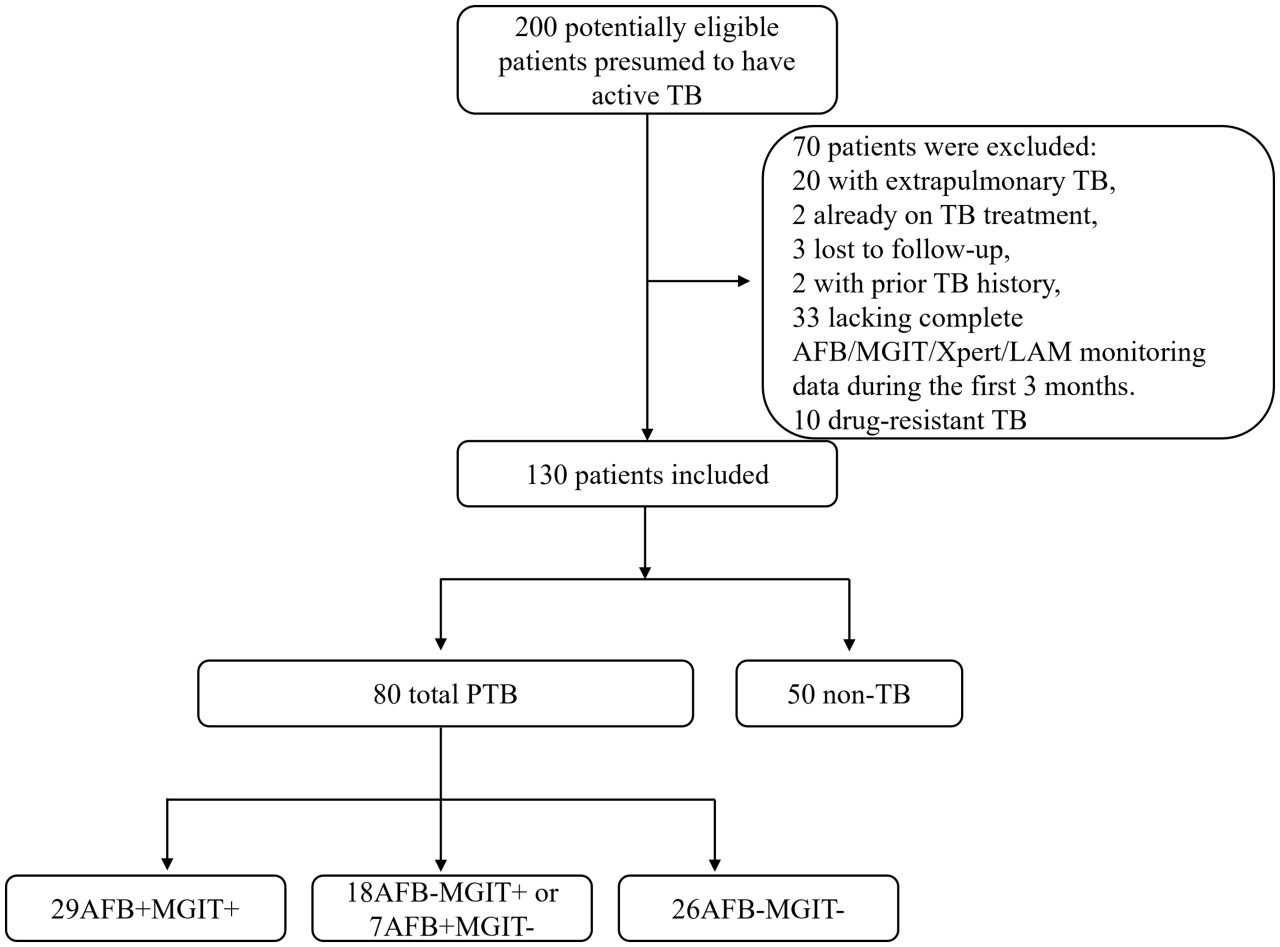
Patient enrollment flow diagram. PTB, Pulmonary Tuberculosis; AFB, Acid-fast bacilli; MGIT, Mycobacteria Growth Indicator Tube.

All 80 PTB patients underwent serial monitoring with AFB, MGIT, GeneXpert, and AIMLAM testing at weeks 4, 8, and 12 of anti-tuberculosis therapy to assess treatment response dynamics (Figure 1).

### 2.2 Diagnostic definitions

The diagnosis of PTB was established according to “Diagnosis for pulmonary tuberculosis (WS 288-2017)” (National Health and Family Planning Commission of the People's Republic of China, 2017; Chun-long et al., 2021). PTB diagnosis integrates bacteriological/molecular biological evidence with epidemiological history, clinical manifestations, chest imaging, auxiliary examinations, and differential diagnosis. Definitive confirmation requires either bacteriological or pathological verification. This study classified PTB patients into:

1 Bacteriologically Confirmed Cases (*n* = 57), meeting ≥1 of the following criteria:
 - AFB smear-positive PTB: ≥2 positive smears OR 1 positive smear with active TB-compatible radiographic lesions OR 1 positive smear with MGIT culture positivity.
 - Culture-positive PTB: Active TB-compatible radiographic lesions with MGIT positivity (without smear confirmation).
 - Molecular test-positive PTB: Active TB-compatible radiographic lesions with GeneXpert MTB/RIF positivity (without smear/culture confirmation).

2 Clinically Diagnosed Cases (*n* = 23), required both:
 - Chest imaging (CT or X-ray) demonstrating active TB-compatible lesions.
 - Plus ≥1 of:
  a) Characteristic TB symptoms.
  b) Tuberculin skin test (TST) positivity (induration ≥10 mm).
  c) Positive interferon-gamma release assay (IGRA).
  d) Positive TB serological antibody test.

Non-TB was defined as patients not eligible for PTB, were defined as individuals who:

- Presented with symptoms clinically suggestive of PTB.
- Underwent comprehensive diagnostic evaluation, were conclusively excluded from PTB diagnosis:

a) Chest imaging, (chest X-ray/CT).
b) Bacteriological testing (AFB smear, MGIT culture, GeneXpert MTB/RIF and others).
c) Diagnostic therapeutic trials with non-TB antimicrobials.

### 2.3 Samples collection and processing

#### 2.3.1 Sputum samples

The collection procedure follows “Diagnosis for pulmonary tuberculosis (WS 288–2017)” (National Health and Family Planning Commission of the People's Republic of China, 2017; Chun-long et al., 2021):

1. Instant sputum refers to specimens expectorated by patients after deep breathing during medical consultation; morning sputum refers to specimens deeply expectorated immediately after waking up in the morning following mouth rinsing with water; night sputum refers to specimens expectorated during the night before submission. Qualified sputum specimens should exhibit purulent, caseous, or mucopurulent characteristics, with a recommended volume of 3 mL to 5 mL.
2. Sputum specimens shall be inspected by laboratory personnel or trained qualified staff. Substandard specimens require re-submission. When qualified specimens are difficult to obtain, bacteriological examination should still be performed, but the specimen characteristics must be documented for reference during result analysis.

#### 2.3.2 Urine samples

Urine sample collection adhered to the following protocol: Each specimen required a minimum volume of 10 mL. Participants were required to provide clean-catch mid-stream urine specimens, with exclusion of samples exhibiting proteinuria, lipiduria, or gross contaminants. Post-collection, samples were immediately refrigerated at 2 °C–8 °C, with mandatory completion of testing within 7 days. Residual urine aliquots were preserved at −20 °C for potential analytical verification. Per manufacturer specifications, frozen specimens underwent ≤3 freeze–thaw cycles when analyzed. For all AIMLAM testing in this study, exclusively fresh urine specimens were utilized, never subjected to freezing.

### 2.4 Urinary LAM detection (chemiluminescence)

For urinary LAM detection, 10 mL of midstream urine was collected from the patient, and the test was performed following the instructions provided by the manufacturer (Gao et al., 2024; Li et al., 2025) (AIMLAM, Guangzhou Leide Bioscience Co., LTD., China). All collected urine samples were centrifuged at 300 × g for 5 min. 4 mL of supernatant were transferred to a new 10 mL centrifuge tube for immediate processing. The residual supernatant was preserved at −20 °C for potential retesting verification. Then, 100 uL of the magnetic bead reagent, which contains LAM-capturing antibodies, was added to the centrifuge tube and mixed. The tube was labeled for identification. The labeled centrifuge tube was placed in a rotating mixer and incubated at room temperature with a rotation speed of 30–50 rpm for 2 h. After incubation, the centrifuge tube was placed on a magnetic rack for adsorption. Once the components were fully separated, the liquid above the sediment was discarded. Resuspend the pellet in 200 μL sample dilution buffer. The mixture was thoroughly mixed using a vortex mixer. Within 5 min, the sample was processed following the operation manual of the LAM detection chemiluminescence analyzer. Process samples using the LAM chemiluminescence analyzer (SMART 500S, KEYSMILE Co., LTD., China) according to manufacturer’s instructions (AIMLAM System, Leide Bioscience). Positive result threshold: LAM concentration ≥0.45 U/mL.

### 2.5 Laboratory methods

Sputum and urine samples were collected at baseline (Pre) and at week 4, 8, and 12 of treatment. Sputum samples were processed for AFB smear microscopy, MGIT culture, and GeneXpert MTB/RIF testing according to standard protocols. Urine samples were processed for LAM detection, using the AIMLAM assay (chemiluminescence-based), following the manufacturer’s instructions. Laboratory personnel remained blinded to clinical diagnoses throughout testing, with exclusive responsibility for technical execution to prevent measurement bias.

AFB Smear Microscopy: Sputum samples were stained using the Ziehl-Neelsen method and examined under a light microscope. Results were graded according to the World Health Organization (WHO) scale (negative, scanty, 1+, 2+, 3+, 4+).

MGIT Culture: Sputum samples were decontaminated using the N-acetyl-L-cysteine–NaOH method and inoculated into MGIT (BD BACTEC MGIT 320, Becton, Dickinson and Company, East Rutherford, USA). Tubes were incubated at 37 °C and monitored for growth using the BACTEC MGIT 960 system. Positive cultures were confirmed by acid-fast staining and MPT64 antigen testing.

GeneXpert MTB/RIF: Sputum samples were processed according to the manufacturer’s protocol (Cepheid, Sunnyvale, CA, USA). The assay simultaneously detects *Mycobacterium tuberculosis* complex DNA and rifampicin resistance-associated mutations in the rpoB gene.

### 2.6 Statistical analysis and sample size

Data analysis was performed using SPSS 20.0 (IBM Corp., USA). Descriptive statistics were reported as mean ± standard deviation (SD) for normally distributed continuous variables and median (interquartile range, IQR) for non-normally distributed variables. Group comparisons for parametric data were conducted using Student’s *t*-test, while non-parametric data were analyzed with the Mann–Whitney U test. Diagnostic performance metrics, including sensitivity, specificity, positive predictive value (PPV), negative predictive value (NPV), and Cohen’s kappa coefficients for inter-method agreement, were calculated against the PTB. Differences in proportions (e.g., sensitivity comparisons) were assessed using chi-square or Fisher’s exact tests as appropriate.

For longitudinal monitoring of treatment response, changes in time to positivity (TTP), AFB smear grades, GeneXpert levels, AIMLAM concentrations, and positivity rates across follow-up timepoints (pre, weeks 4, 8, 12) were evaluated using paired *t*-tests (parametric) or Wilcoxon signed-rank tests (non-parametric). Receiver operating characteristic (ROC) curves were generated to evaluate the predictive performance of baseline LAM levels (PreLAM), ΔLAM (PreLAM concentration - Week 4 LAM concentration), and the combined PreLAM+ΔLAM model for treatment-induced LAM conversion. For the combined PreLAM+ΔLAM model, both biomarkers were first incorporated into a binary logistic regression analysis to derive a composite predictor score. This integrated score was subsequently used to generate the combined ROC curve, enabling assessment of its prognostic value for conversion to culture negativity. Optimal cutoff values were determined using Youden’s index. Additionally, this integrated score was applied in subsequent Kaplan–Meier (KM) analyses and Cox analyses. Survival analyses for conversion time (time to negativity) were performed using Kaplan–Meier curves with log-rank tests, and Cox proportional hazards regression models (univariate and multivariate) were applied to identify predictors of LAM conversion. Hazard ratios (HRs) with 95% confidence intervals (CIs) were reported.

The prevalence of tuberculosis in the target population is 60%. Literature review indicates that the sensitivity of the gold standard sputum culture ranges from 36 to 46%, with specificity between 95 and 100%. According to published studies (Gao et al., 2024; Li et al., 2025; Huang et al., 2023), AIMLAM demonstrates sensitivity of 51–55% and specificity of 95–100%. With *α* = 0.05 and *β* = 0.2, PASS software was used for sample size calculation, yielding a minimum requirement of 115 participants: 69 PTB cases and 46 non-TB cases.

Data visualization was performed using specialized software: heatmaps and bubble plots were generated in OriginPro 2022 (OriginLab, USA), dot/bar plots in GraphPad Prism 9.0 (GraphPad Software, USA), and Kaplan–Meier/ROC curves in SPSS. All statistical tests were two-tailed, with *p* < 0.05 considered significant.

### 2.7 Ethical considerations

This study adhered to the Helsinki Declaration and was approved by the Medical Ethics Committee of Ningbo Yinzhou No.2 Hospital (approval number: 2025009). Written informed consent was obtained from all participants prior to enrollment. Patient confidentiality was maintained throughout the study, and all data were anonymized for analysis.

## 3 Results

### 3.1 Patient demographic characteristics

Baseline characteristics revealed no significant differences between the PTB group and the non-TB group in terms of age, gender, BMI, smoking status, drinking status, and comorbidities (including diabetes and hepatitis B) (all *p* > 0.05). In laboratory tests, the PTB group showed significantly lower CRP levels compared to the non-TB group (1.00 [0.00, 18.70] vs. 1.15 [0.00, 20.48], *p* = 0.003) (Table 1). Other laboratory indicators, such as WBC, neutrophil count, monocyte count, ESR, albumin, and prealbumin, showed no significant differences between the two groups (all *p* > 0.05) (Table 1).

**Table 1:** Patient demographic characteristics.

Characteristics	PTB (*n* = 80)	Non-TB (*n* = 50)	*p* value
Age (years, mean±SD)	44.59 ± 19.55	51.21 ± 17.72	0.117
Gender [male *n* (%)]	61 (76.25%)	30 (60%)	0.049
BMI (kg/m^2^, mean±SD)	21.93 ± 3.76	21.87 ± 3.50	0.950
smoker [yes, *n* (%)]	24 (30.00%)	10 (20%)	0.207
Drinker [yes, *n* (%)]	11 (13.75%)	5 (10%)	0.527
Comorbidities			
Diabetes [yes, *n* (%)]	10 (12.50%)	2 (4.00%)	0.103
Hepatitis B [yes, *n* (%)]	3 (3.75%)	2 (4.00%)	0.943
Laboratory test			
WBC (×10^9^/L, mean±SD)	6.59 ± 2.26	6.74 ± 3.87	0.830
Neutrophil (×10^9^/L, mean±SD)	4.45 ± 2.03	4.82 ± 3.86	0.573
Lymphocyte (×10^9^/L, mean±SD)	1.44 ± 0.56	1.23 ± 0.52	0.158
Monocyte [×10^9^/L, median (IQR)]	0.50 [0.36, 0.65]	0.51 [0.35, 0.66]	0.790
CRP [mg/L, median (IQR)]	1.00 [0.00, 18.70]	1.15 [0.00. 20.48]	0.003
ESR (mm/h, mean±SD)	19.71 ± 21.25	17.71 ± 13.34	0.817
Albumin (g/L, mean±SD)	41.09 ± 4.55	41.69 ± 6.94	0.677
Prealbumin (mg/L, mean±SD)	216.32 ± 72.04	214.21 ± 90.06	0.923

BMI, Body Mass Index (kg/m^2^); PTB, Pulmonary tuberculosis; WBC, White Blood Cell Count (×10^9^/L); CRP, C-Reactive Protein (mg/L); ESR, Erythrocyte sedimentation rate.

### 3.2 Diagnostic performance of methods

AIMLAM demonstrated a sensitivity of 58.75%, slightly higher than AFB smear (45.00%, *p* = 0.082) and similar to MGIT culture (58.75%, *p* = 1.00), but slightly lower than GeneXpert (61.25%, *p* = 0.205). Conventional methods (AFB, MGIT, and GeneXpert) maintained a specificity of 100%, compared with 96.67% for AIMLAM. All comparator methods achieved 100% PPV, whereas AIMLAM showed a PPV of 97.92%. The NPVs were 53.19, 60.24, 61.73, and 59.29% for AFB smear, culture, GeneXpert, and AIMLAM, respectively. The kappa coefficients for diagnostic agreement with PTB were 0.386, 0.523, 0.549, and 0.490 for AFB, MGIT, GeneXpert, and AIMLAM, respectively (Table 2). When evaluated against bacteriologically confirmed PTB, AIMLAM showed sensitivity of 75.44%, specificity of 91.78%, PPV of 87.76%, and NPV of 82.72% (kappa = 0.683) (Supplementary Table 1).

**Table 2:** Diagnostic performance of different methods for tuberculosis.

Methods		PTB		Sensitivity [95% CI]	*p* value (sensitivity)	Specificity [95% CI]	PPV [95% CI]	NPV [95% CI]	Kappa value
		positive	negative						
AFB	Positive	36	0	45.00% [34.00, 56.49%]	0.082	100.00% [91.12, 100%]	100.00% [87.99, 100%]	53.19% [42.66, 63.46%]	0.386
	Negative	44	50						
MGIT	Positive	47	0	58.75% [47.19, 69.47%]	1.0	100.00% [91.11, 100%]	100.00% [90.59, 100%]	60.24% [48.89, 70.64%]	0.523
	Negative	33	50						
GeneXpert	Positive	49	0	61.25% [49.67, 71.74%]	0.205	100.00% [91.11, 100%]	100.00% [90.94, 100%]	61.73% [50.22, 72.11%]	0.549
	Negative	31	50						
AIMLAM	Positive	47	2	58.75% [47.19, 69.47%]	-	96.00% [85.14, 99.30%]	95.92% [84.86, 99.29%]	59.26% [47.46, 69.87%]	0.490
	Negative	33	48						

PTB, Pulmonary tuberculosis; PPV, Positive predictive value; NPV, Negative predictive value; AFB, Acid-fast bacilli; MGIT, Mycobacteria Growth Indicator Tube.

### 3.3 Early monitoring performance of methods

**Figure 2:**
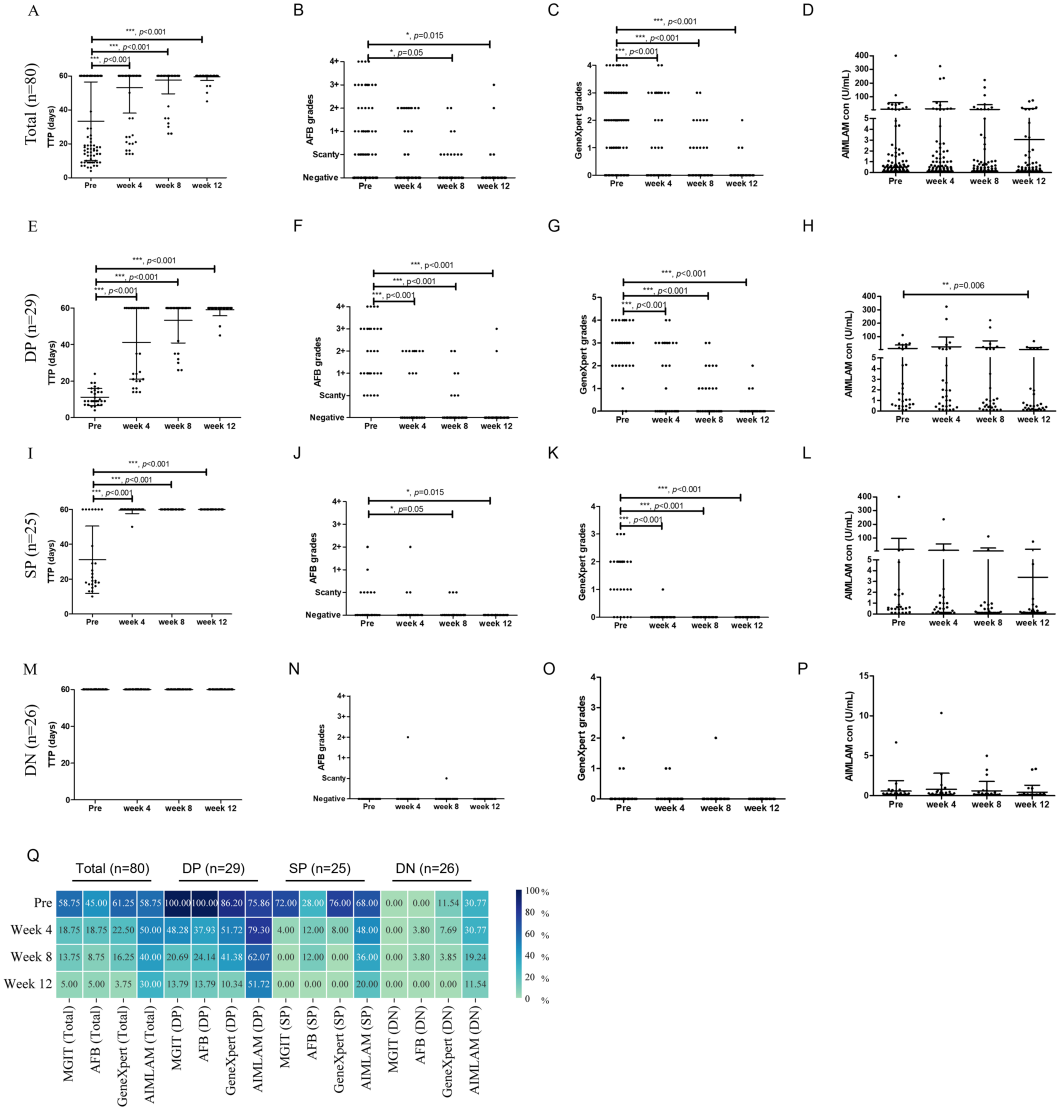
Longitudinal monitoring of tuberculosis treatment response by diagnostic methods. Total cohort dynamics of **(A)** MGIT time to positivity, **(B)** AFB smear grades, **(C)** GeneXpert grades, and **(D)** AIMLAM concentration. **(E–H)** DP group dynamics for **(E)** TTP, **(F)** AFB, **(G)** GeneXpert, and **(H)** AIMLAM. **(I–L)** SP group dynamics for **(I)** TTP, **(J)** AFB, **(K)** GeneXpert, and **(L)** AIMLAM. **(M–P)** DN group dynamics for **(M)** TTP, **(N)** AFB, **(O)** GeneXpert, and **(P)** AIMLAM. **(Q)** Longitudinal positivity rates of four diagnostic methods. Paired *t*-test for semi-quantitative data; Chi-square test for percentage comparisons. *, *p* < 0.05; **, *p* < 0.01; ***, *p* < 0.001; MGIT, Mycobacteria Growth Indicator Tube; AFB, Acid-fast bacilli smear microscopy; TTP, Time to positivity of MGIT; DP, Double-positive (AFB+/MGIT+); SP, Single-positive (AFB+/MGIT− or AFB−/MGIT+); DN, Double-negative (AFB−/MGIT−); PreLAM, baseline LAM.

In the total cohort, MGIT TTP increased significantly from the first treatment month (week 4) (*p* < 0.01, Figure 2A), accompanied by reductions in GeneXpert semi-quantitative levels (*p* < 0.01, Figure 2C), while AFB grades and AIMLAM concentrations showed no significant decrease (Figures 2B,D). Stratified analysis by MGIT/AFB status revealed: In the DP group, MGIT TTP significantly increased from the first treatment month (week 4) (Figure 2E), paralleled by reductions in AFB and GeneXpert grades (Figures 2F,G), whereas AIMLAM concentrations showed significant decline only at week 12 (Figure 2H). MGIT/AFB/GeneXpert exhibited maximal positivity rate reduction at week 4 (Δ = 34.48–62.07%), declining to 10.34–13.79% by week 12, while AIMLAM positivity decreased gradually from week 8, maintaining 51.72% at week 12 (Figure 2Q).

In the SP group, significant reductions in MGIT TTP and GeneXpert grades occurred at week 4 (Figures 2I,K), with delayed AFB decline until week 8 (Figure 2J), yet AIMLAM concentrations showed no significant reduction through week 12 (Figure 2L). The positivity rates of MGIT/AFB/GeneXpert plummeted to 4–12% at week 4 versus AIMLAM’s gradual decrease to 20% by week12 (Figure 2Q). The DN group demonstrated baseline positivity of 11.54% for GeneXpert and 30.77% for AIMLAM. Pretreatment positivity declined from 11.54 to 0% (week 4-week 12) for GeneXpert, compared to AIMLAM’s decrease from 30.77 to 11.54% (Figure 2Q). Semi-quantitative results for MGIT/AFB/GeneXpert/AIMLAM remained at low levels throughout follow-up (Figures 2M–P).

### 3.4 The diagnostic-monitoring dual-dimensional performance

**Figure 3:**
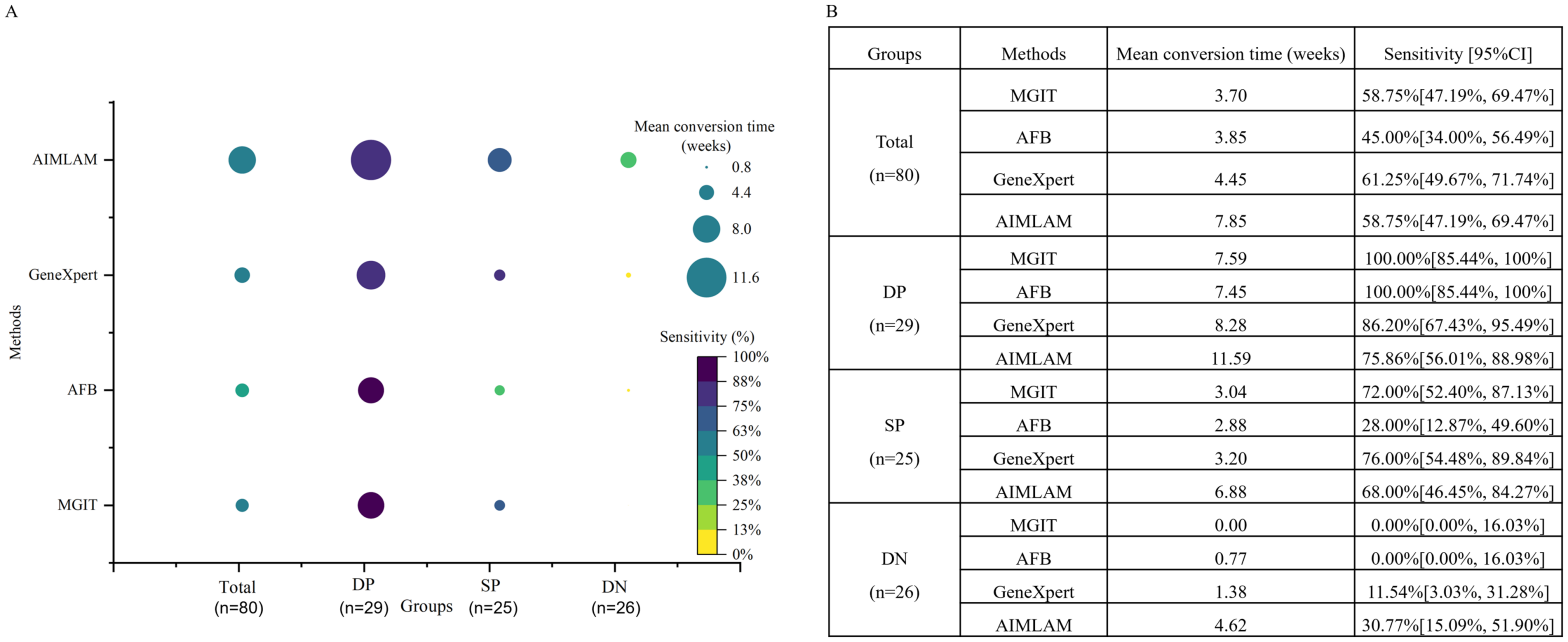
Diagnostic-monitoring dual-dimensional performance of four methods. **(A)** Bubble plot illustrating the dual-dimensional performance of diagnostic methods, with sensitivity (color gradient) reflecting diagnostic accuracy and time to conversion (bubble size) indicating monitoring utility (longer time = superior monitoring). **(B)** Sensitivity and mean time to conversion by method and groups. MGIT, Mycobacteria Growth Indicator Tube; AFB, Acid-fast bacilli smear microscopy; DP, Double-positive (AFB+/MGIT+); SP, Single-positive (AFB+/MGIT− or AFB−/MGIT+); DN, Double-negative (AFB−/MGIT−).

In this study, sensitivity was considered as one key indicator reflecting diagnostic performance (in the context of comparable specificity), while longer conversion time (time to negative result) was regarded as a potential marker suggesting greater utility in treatment monitoring. In the total cohort, MGIT exhibited moderate diagnostic sensitivity (57.85%) and shortest conversion time (3.7 weeks). AFB achieved lowest diagnostic sensitivity (45.00%) with a conversion time of 3.85 weeks. GeneXpert exhibited the best sensitivity (75%) with a conversion time of 4.45 weeks. AIMLAM demonstrated balanced diagnostic-monitoring utility with a sensitivity of 58.75% and the longest mean conversion time (7.85 weeks). For DP group, AFB and MGIT showed optimal diagnostic sensitivity (100%) but limited monitoring utility (7.45–7.59 weeks). GeneXpert demonstrated moderate sensitivity (86.20%) and a slightly longer conversion time (8.28 weeks), while AIMLAM, despite its lower diagnostic sensitivity (75.86%), exhibited the longest conversion time (11.59 weeks). For SP group, AFB failed diagnostically (28% sensitivity) and rapid conversion (2.88 weeks), while MGIT showed 72% sensitivity with a comparable conversion time (3.04 weeks). GeneXpert achieved higher sensitivity (76%) but shorter conversion time (3.2 weeks). AIMLAM balanced moderate diagnostic sensitivity (68%) with the best monitoring performance (6.88 weeks). For DN group, all methods performed poorly, yet AIMLAM retained dual advantages: highest sensitivity (30.77%) and longest conversion time (4.62 weeks). GeneXpert exhibited marginally better sensitivity (11.54%) and conversion time (11.54 weeks) than AFB/MGIT (0%, 1–1.77 weeks) (Figure 3).

### 3.5 Predictive performance of PreLAM and ΔLAM

**Figure 4:**
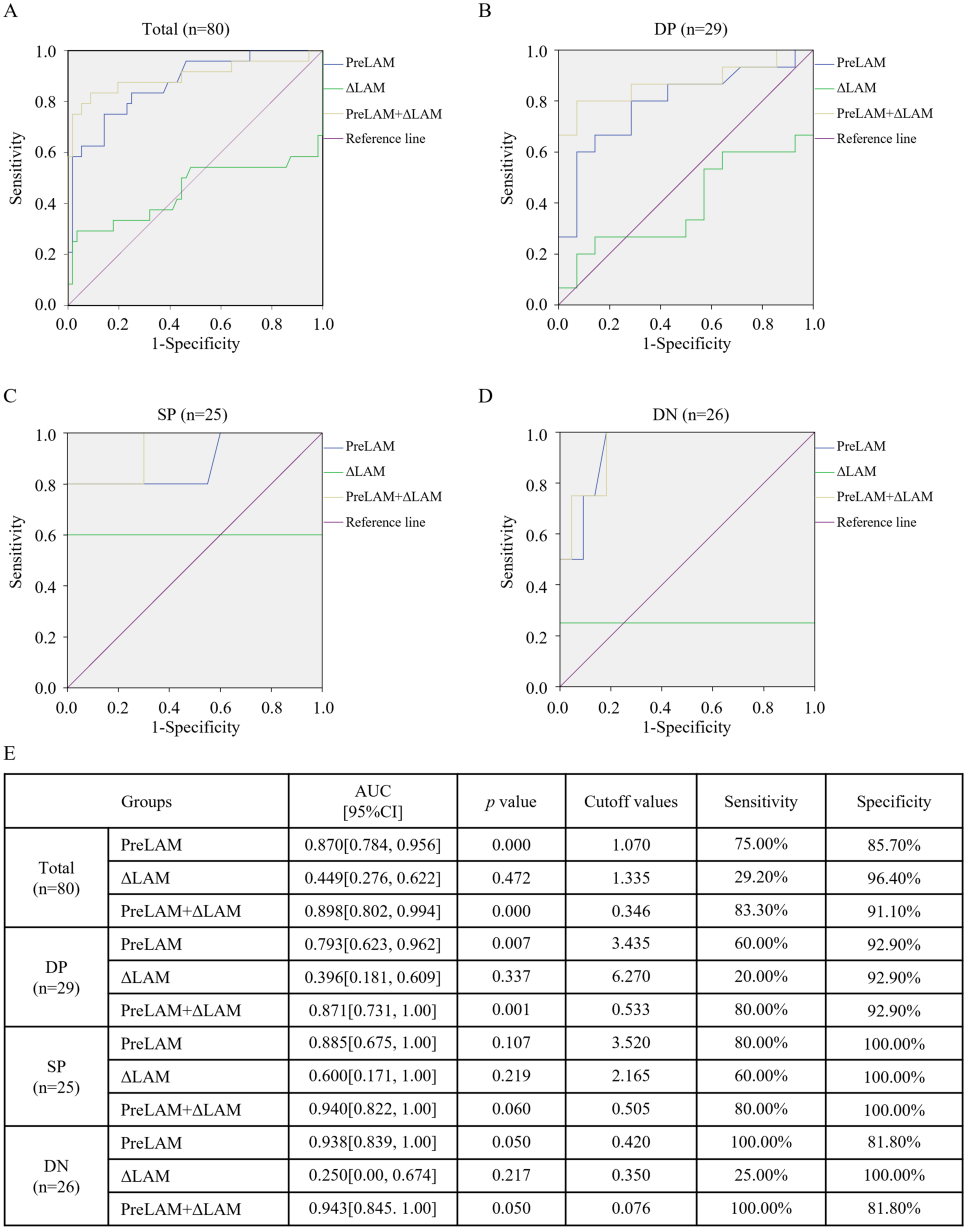
ROC analysis of PreLAM, ΔLAM, and their combined model for predicting LAM conversion to negativity after treatment. ROC curves comparing baseline urine LAM (PreLAM), week 4 LAM change (ΔLAM, PreLAM- Week 4 LAM), and the combined model (PreLAM+ΔLAM) in **(A)** total group, **(B)** DP group, **(C)** SP group, **(D)** DN group. **(E)** Summary of predictive performance metrics across all groups. MGIT, Mycobacteria Growth Indicator Tube; AFB, Acid-fast bacilli smear microscopy; TTP, Time to positivity of MGIT; DP, Double-positive (AFB+/MGIT+); SP, Single-positive (AFB+/MGIT− or AFB−/MGIT+); DN, Double-negative (AFB−/MGIT−); PreLAM, baseline LAM; ΔLAM, PreLAM- Week 4 LAM.

ROC curve analysis of baseline PreLAM levels, ΔLAM (Week 4 LAM change), and their combined model (PreLAM+ΔLAM) revealed that the combine model demonstrated the highest predictive performance for LAM conversion to negativity after treatment across all groups, achieving the maximum AUC values (Total: 0.898, *p* < 0.001; DP: 0.871, *p* = 0.001; SP: 0.940, *p* = 0.06; DN: 0.943, *p* = 0.05) and optimal sensitivity (80.0–100%) while maintaining high specificity (81.8–100%), consistently outperforming standalone PreLAM (AUC = 0.87–0.938) and ΔLAM (AUC = 0.25–0.6) (Figure 4).

### 3.6 Risk factor analysis for LAM conversion

**Figure 5:**
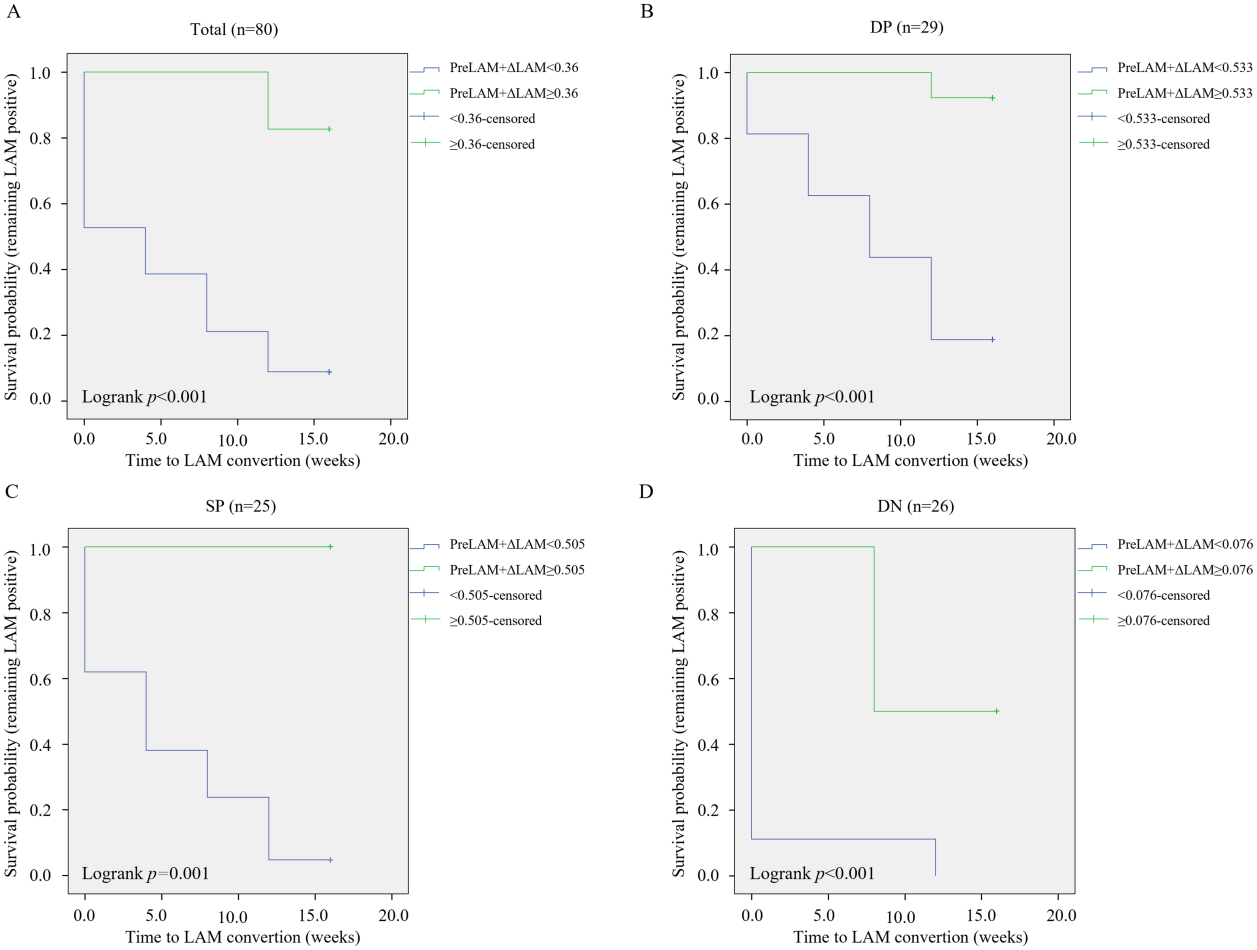
Kaplan–Meier survival curves for LAM conversion. Stratified by PreLAM+ΔLAM levels in **(A)** total cohort, **(B)** Double-positive (DP: AFB+/MGIT+) group, **(C)** Single-positive (SP: AFB+/MGIT− or AFB−/MGIT+) group, **(D)** Double-negative (DN: AFB−/MGIT−) group. DP, Double-positive (AFB+/MGIT+); SP, Single-positive (AFB+/MGIT− or AFB−/MGIT+); DN, Double-negative (AFB−/MGIT−); PreLAM, baseline LAM; ΔLAM, PreLAM- Week 4 LAM.

Kaplan–Meier analysis demonstrated significantly accelerated LAM conversion after 3-month anti-TB treatment in patients with low PreLAM+ΔLAM values across all cohorts (*p* < 0.001 for total/double-positive/double-negative groups; *p* = 0.001 for single-positive group) (Figure 5). Univariate Cox regression identified albumin (HR = 1.079, *p* = 0.021), prealbumin (HR = 1.005, *p* = 0.007), AFB smear grade (HR = 0.680, *p* < 0.001), and PreLAM+ΔLAM (HR = 0.006, *p* < 0.001) as key predictors in the total cohort. In double-positive cohort, AFB grade (HR = 0.571, *p* = 0.016) and PreLAM+ΔLAM (HR = 0.013, *p* = 0.002) as key predictors. In Single-positive cohort, gender (HR = 0.972, *p* = 0.025) and PreLAM concentration (HR = 0.404, *p* = 0.032) as key predictors. In double-negative, PreLAM (HR = 0.111, *p* = 0.035) and PreLAM+ΔLAM (HR = 0.026, *p* = 0.045) as key predictors (Table 3). Multivariate analysis confirmed PreLAM+ΔLAM as an independent predictor in the total cohort (HR = 0.013, *p* = 0.001) and double-positive cohort (HR = 0.020, *p* = 0.006), while PreLAM remained protective in single-positive patients (HR = 0.411, *p* = 0.040). No significant predictors persisted in the double-negative cohort after adjustment (Table 4).

**Table 3:** Univariate cox regression analysis.

Characteristics	Univariate cox analyses							
	Total group (*n* = 80)		Double positive group (*n* = 29)		Single positive group (*n* = 25)		Double negative group (*n* = 26)	
	HR [95% CI]	*p*	HR [95% CI]	*p*	HR [95% CI]	*p*	HR [95% CI]	*p*
Age	0.986 [0.973, 1.00]	0.053	1.253 [0.349, 4.491]	0.73	0.972 [0.949, 0.996]	0.025	0.995 [0.976, 1.013]	0.565
Gender (male vs. female)	0.917 [0.501, 1.679]	0.779	0.982 [0.950, 1.016]	0.296	0.56 [0.201, 1.560]	0.267	1.04 [0.407, 2.662]	0.934
BMI	0.993 [0.925–1.065]	0.843	0.945 [0.821, 1.088]	0.43	1.039 [0.918, 1.176]	0.545	1.032 [0.906, 1.175]	0.635
Smoker (yes vs. no)	1.082 [0.612–1.913]	0.786	1.073 [0.336, 3.428]	0.905	0.983 [0.388, 2.488]	0.971	1.142 [0.445, 2.930]	0.782
Drinking (yes vs. no)	1.462 [0.713–2.997]	0.3	0.750 [0.098, 5.739]	0.782	1.476 [0.473, 4.6]	0.502	1.5 [0.500, 4.499]	0.469
Comorbidities								
Previous TB history (yes vs. no)	0.971 [0.387–2.434]	0.95	2.064 [0.458, 9.298]	0.345	0.302 [0.04, 2.272]	0.245	1.714 [0.390, 7.543]	0.476
Diabetes (yes vs. no)	0.397 [0.143–1.101]	0.076	0.221 [0.029, 1.694]	0.146	0.817 [0.239, 2.793]	0.747	/	/
Laboratory test								
WBC	0.961 [0.850–1.088]	0.531	1.049 [0.882, 1.248]	0.59	0.936 [0.720, 1.218]	0.625	1.039 [0.806, 1.341]	0.766
Neutrophil	0.950 [0.822–1.097]	0.483	1.080 [0.884, 1.319]	0.451	0.915 [0.670, 1.248]	0.574	1.023 [0.789, 1.327]	0.863
Lymphocyte	1.182 [0.766–1.824]	0.451	0.686 [0.220, 2.139]	0.516	1.664 [0.496, 5.582]	0.41	1.055 [0.623, 1.786]	0.842
Monocyte	1.081 [0.729–1.604]	0.699	1.173 [0.243, 5.656]	0.842	1.199 [0.844, 1.704]	0.31	0.947 [0.077, 11.695]	0.966
CRP	0.992 [0.979–1.006]	0.256	1.005 [0.989, 1.022]	0.517	0.997 [0.971, 1.023]	0.807	0.983 [0.947, 1.020]	0.365
Albumin	1.079 [1.011–1.151]	0.021	1.005 [0.919, 1.168]	0.566	1.148 [0.984, 1.34]	0.08	1.027 [0.931, 1.132]	0.596
Prealbumin	1.005 [1.001–1.009]	0.007	1.003 [0.995, 1.011]	0.426	1.005 [0.997, 1.014]	0.199	1.003 [0.998, 1.008]	0.3
AFB smear gradings	0.68 [0.550–0.841]	<0.001	0.571 [0.362, 0.901]	0.016	0.741 [0.395, 1.398]	0.35	0.638 [0.205, 1.983]	0.437
MGIT TPP	1.013 [1.001–1.024]	0.027	1.012 [0.906, 1.131]	0.829	0.988 [0.964, 1.013]	0.347		
GeneXpert grading	0.738 [0.607–0.899]	0.002	0.727 [0.470, 1.125]	0.152	0.988 [0.642, 1.519]	0.954		/
PreLAM concentration	0.875 [0.773–0.989]	0.033	0.951 [0.891, 1.015]	0.13	0.404 [0.177, 0.923]	0.032	0.111 [0.014, 0.856]	0.035
ΔLAM concentration	1.000 [0.996–1.003]	0.896	1.011 [0.991, 1.031]	0.294	0.878 [0.705, 1.095]	0.248	2.761 [0.804, 9.478]	0.107
PreLAM+ΔLAM	0.006 [0.001–0.062]	<0.001	0.013 [0.001, 0.208]	0.002	0.001 [0.000, 1.116]	0.054	0.026 [0.001, 0.928]	0.045

BMI, Body Mass Index (kg/m^2^); TB, Tuberculosis; WBC, White Blood Cell Count (×10^9^/L); CRP, C-Reactive Protein (mg/L); MGIT, Mycobacteria Growth Indicator Tube; AFB, Acid-fast bacilli smear microscopy; TTP, Time to positivity of MGIT; DP, Double-positive (AFB+/MGIT+); SP, Single-positive (AFB+/MGIT− or AFB−/MGIT+); DN, Double-negative (AFB−/MGIT−); PreLAM, baseline LAM. ΔLAM, week 4 LAM change.

**Table 4:** Multivariate cox regression analysis.

Characteristics	Multivariate cox analyses							
	Total group (*n* = 80)		Double positive group (*n* = 29)		Single positive group (*n* = 25)		Double negative group (*n* = 26)	
	HR [95% CI]	*p*	HR [95% CI]	*p*	HR [95% CI]	*p*	HR [95% CI]	*p*
Age					[0.963–1.011]	0.283		
Albumin	0.979 [0.897–1.068]	0.637						
Prealbumin	1.001 [0.996–1.006]	0.694						
AFB smear gradings	0.859 [0.592–1.247]	0.424	0.73 [0.458–1.164]	0.186				
MGIT TPP	1.004 [0.984–1.024]	0.719						
GeneXpert grading	1.093 [0.683–1.751]	0.71						
PreLAM concentration	0.991 [0.935–1.051]	0.771			0.411 [0.176–0.962]	0.04	0.157 [0.001–17.831]	0.443
PreLAM+ΔLAM	0.013 [0.001–0.149]	0.001	0.02 [0.001–0.331]	0.006			0.534 [0.000–1372.51]	0.875

MGIT, Mycobacteria Growth Indicator Tube; AFB, Acid-fast bacilli smear microscopy; TTP, Time to positivity of MGIT; DP, Double-positive (AFB+/MGIT+); SP, Single-positive (AFB+/MGIT− or AFB−/MGIT+); DN, Double-negative (AFB−/MGIT−); PreLAM, baseline LAM. ΔLAM, week 4 LAM change.

## 4 Discussion

The proactive identification of cases can reduce the transmission of tuberculosis, which necessitates the development of simpler and faster technologies to assist clinics in detecting more tuberculosis patients at an earlier stage, thereby curbing the spread of the disease (Ivanyi, 2014). This study provides a comprehensive evaluation of AIMLAM’s diagnostic and monitoring performance compared to conventional TB diagnostic methods (AFB smear, MGIT culture, and GeneXpert), while introducing a novel predictive model (PreLAM+ΔLAM) for early treatment response. The findings highlight AIMLAM’s unique dual utility in TB management—balancing diagnostic sensitivity with prolonged monitoring capability—and establish the clinical relevance of LAM antigen dynamics as a predictive biomarker for early treatment response.

AIMLAM demonstrated superior sensitivity (58.75%) to AFB smear (45.00%), aligning closely with MGIT culture (58.75%) and GeneXpert (61.25%), albeit with slightly lower specificity (96.67% vs. 100% for conventional methods). These findings are consistent with previously reported results demonstrating 51–55% sensitivity, significantly higher than AFB smear and comparable to culture-based sensitivity (Gao et al., 2024; Li et al., 2025; Huang et al., 2023). The present study found that in the Total, DP, and SP groups, GeneXpert exhibited marginally higher positive detection rates than AIMLAM before treatment (Pre) (61.25% vs. 58.75, 86.2% vs. 75.86, and 76% vs. 68%, respectively). However, in the DN group, AIMLAM showed a slightly higher rate than GeneXpert (30.77% vs. 11.54%). As a molecular diagnostic technique, GeneXpert typically achieves high sensitivity and specificity (Acharya et al., 2020). Its lower positive detection rate in the DN group may be attributed to the low bacterial load in samples (Hai et al., 2019) or poor sputum quality (Mulengwa et al., 2022), which could lead to false-negative results. This positions AIMLAM as a viable alternative in settings where rapid smear microscopy lacks sensitivity, and it holds advantages for populations with scanty sputum or difficulties in sputum collection, such as children and patients with extrapulmonary infections (Huang et al., 2023).

However, its most striking feature lies in its monitoring performance (Peter et al., 2016). While conventional methods (AFB, MGIT, GeneXpert) showed rapid declines in positivity rates and semi-quantitative levels within the first month of treatment—reflecting early bacterial load reduction—AIMLAM’s antigen concentrations decreased gradually, with significant reductions only at week 12 in the DP group. This delay may be attributed to: 1. Continuous LAM release from macrophage-engulfed bacilli undergoing lysosomal degradation (Ghazaei, 2018) and sterilizing granulomas where residual cell-wall fragments accumulate (Davuluri et al., 2023); 2. Potential undetected drug-resistant cases with suboptimal treatment response, despite rigorous exclusion of confirmed resistant tuberculosis through pre-enrollment (Italia et al., 2023). However, it should be emphasized that the clinical utility of LAM-based assays for monitoring treatment efficacy in EPTB remains unexplored, as no relevant studies have been reported to date.

GeneXpert exhibited the highest diagnostic sensitivity (61.25%), which is consistent with previous studies (Burger et al., 2022; Huang et al., 2023). However, its shorter conversion time (4.45 weeks vs. AIMLAM’s 7.85 weeks in the total cohort) underscores its limitation in long-term monitoring. Notably, a recent study reported that 80% of patients with AFB/MGIT/Xpert/MBLA quadruple-positive pulmonary TB remained positive by these methods at week 14 of treatment (Neumann et al., 2025), contrasting sharply with the 10.34% positivity rate observed in our DP group at week 12. This discrepancy may stem from differences in study populations: while the cited study enrolled patients uniformly positive across all four methods (AFB/MGIT/Xpert/MBLA), our DP group included only AFB/MGIT double-positive cases (*n* = 29 in both cohorts). The stricter inclusion criteria in the former—requiring positivity across sputum-dependent and molecular assays—likely selected for patients with higher baseline bacterial burdens, explaining the slower clearance rates (Neumann et al., 2025). Conversely, AIMLAM’s prolonged antigen detection window could fill a critical gap in evaluating end-of-treatment outcomes, particularly in high-burden DP patients, where its conversion time (11.59 weeks) far exceeded other methods.

Our study found that AIMLAM exhibited a specificity of 95.92% in non-TB controls, which was lower than that of AFB smear, MGIT culture, and Xpert MTB/RIF (all 100%). This observation aligns with findings from prior research (Huang et al., 2023). The slightly reduced specificity could potentially be explained by several factors: 1. Given the structural homology of LAM across mycobacterial species, the two false-positive cases might be attributed to either latent nontuberculous mycobacteria (NTM) contamination during sample processing—considering the abundance of NTM in the environment (Sharma and Upadhyay, 2020)—or subclinical NTM infection that escaped detection by routine diagnostic approaches. Huang et al. (2023) reported an AIMLAM positivity rate of 36.36% in confirmed NTM patients. Notably, values within the “gray zone” of the assay (i.e., ±15% of the cutoff) are inherently prone to measurement variability. One false-positive case, with a value of 0.50 U/mL relative to the 0.45 U/mL cutoff, falls precisely within this marginal range, which could further contribute to the observed result.

A critical advantage of AIMLAM lies in its non-sputum-dependent design. Unlike AFB smear, MGIT culture, and GeneXpert—which rely heavily on high-quality sputum samples and are thus limited in paucibacillary, pediatric, or extrapulmonary cases (Tendolkar et al., 2021), AIMLAM utilizes urine, a universally accessible specimen (Bulterys et al., 2019). This eliminates challenges associated with sputum collection (e.g., induction difficulties, contamination risks) and expands diagnostic access to underserved populations, including HIV-negative individuals with atypical presentations. Tuberculosis antibodies, including specific antibodies targeting LAM antigens, show promising potential in the diagnosis and therapeutic monitoring of tuberculosis (Bothamley et al., 1992). Ivanyi et al. found that antibody production is associated with the bacterial load of the disease, and antibodies have significantly higher sensitivity in detecting sputum smear-positive pulmonary diseases (Ivanyi, 2012). It has also been shown that antibody levels may increase during anti-tuberculosis treatment. Bothamley et al. observed that the antibody to the ML34 epitope of lipoarabinomannan consistently showed a single peak in antibody levels at 3 to 4 months (Bothamley, 2004). Analyzing the combined value of LAM antigens and antibodies in the diagnosis and therapeutic monitoring of tuberculosis may lead to new discoveries. However, due to the small number of tuberculosis antibody tests conducted in our hospital, they were not included in this study.

The combined PreLAM+ΔLAM model emerged as a novel and robust predictor of early LAM clearance, achieving AUC values >0.87 across all cohorts. Our Kaplan–Meier analysis revealed that low PreLAM+ΔLAM levels were strongly associated with accelerated LAM conversion across all patient cohorts. Notably, PreLAM+ΔLAM emerged as the most robust independent predictor in univariate and multivariate models for the total and double-positive cohorts. To our knowledge, no prior studies have specifically reported predictive models for short-term LAM conversion dynamics during anti-TB therapy. Baseline PreLAM levels likely reflect initial antigen burden and bacterial load, as supported by studies demonstrating correlations between urinary LAM concentrations and baseline sputum mycobacterial load in pulmonary TB (Paris et al., 2017). Meanwhile, ΔLAM (week 4 LAM change) captures early treatment response dynamics, with its magnitude potentially modulated by drug efficacy, host immune status (e.g., CD4 + T-cell function) (Correia-Neves et al., 2019), and metabolic clearance rates (Lawn et al., 2009; Wood et al., 2012). Clinically, this model enables risk stratification: patients with high PreLAM and stagnant ΔLAM may benefit from extended therapy or adjunctive interventions (e.g., immunomodulators), whereas those with low PreLAM and rapid ΔLAM decline could qualify for shortened regimens, reducing overtreatment.

The univariate Cox regression identified albumin and prealbumin as significant predictors in the total cohort, suggesting that nutritional status and systemic inflammation may play pivotal roles in determining treatment outcomes.

Some literature indicates that a too-low prealbumin level can assist in differentiating active tuberculosis from latent tuberculosis infection (Luo et al., 2021), and it has the potential to serve as an indicator for monitoring the therapeutic effect of tuberculosis treatment (Luo et al., 2013). Similarly, AFB smear grade, MGIT TPP, GeneXpert grade inversely correlated with LAM conversion rates, consistent with studies showing that high bacterial load predicts delayed culture conversion (Magombedze et al., 2021).

This study has several limitations. First, this study with an overall sample size of 130, stratifying into key subgroups (80 PTB vs. 50 non-TB; and finer divisions like 29 AFB+/MGIT+, 18 AFB-/MGIT+, 7 AFB+/MGIT-, 26 AFB-/MGIT-) drastically reduces the number of participants within each comparison group. This significantly diminishes statistical power, severely constraining our ability to conduct robust comparisons of methodological sensitivity and specificity across subgroups. Consequently, the risk of failing to detect statistically significant differences is heightened, and the reliability of estimates derived from smaller subgroups is reduced.

Second, the single-center design introduces potential biases related to local treatment protocols and patient population characteristics, which may limit the external validity and generalizability of the findings to broader or diverse healthcare settings and populations. Third, the 12-week observation period precludes evaluation of AIMLAM’s correlation with tuberculosis (TB) relapse or sustained cure (requiring ≥6 months of follow-up), and there is a lack of direct validation using clinical endpoints such as treatment success rates or mortality. Fourth, the current dataset has not yet integrated host immune biomarkers (e.g., cytokines, transcriptomics, CD4+/CD8 + ratios) to elucidate mechanisms underlying LAM dynamics, despite existing evidence showing LAM levels are closely associated with host immune status (e.g., delayed antigen clearance in HIV-coinfected individuals due to immunosuppression) (Wood et al., 2012; Flores et al., 2021). Future work will address these gaps through:

1. Large-scale, multicenter studies to enhance statistical power and generalizability; 2. Extended follow-up periods incorporating treatment completion and monitoring patients for ~1 year post-treatment success to capture relapse data; and 3. Integration of host immune parameters with the PreLAM+ΔLAM model. These steps aim to enable more precise TB management within clinical decision support systems.

## 5 Conclusion

This study positions urinary LAM as a pivotal dual-purpose biomarker in TB care, addressing unmet needs in both diagnosis and therapeutic monitoring. While demonstrating diagnostic performance comparable to established microbiological and molecular methods, LAM’s distinctive strength lies in its capacity to track treatment response beyond the early bactericidal phase. Unlike conventional sputum-dependent assays that rapidly decline in positivity, LAM’s prolonged detection window captures persistent antigen dynamics, reflecting residual bacillary activity—a critical advance for evaluating sustained therapeutic efficacy.

The integration of baseline antigen levels and early-treatment kinetic changes into a predictive model for early treatment response enables early identification of patients potentially requiring closer monitoring or therapy optimization during the initial treatment phase. This approach aligns with precision medicine paradigms, offering actionable insights for personalized intervention strategies early in the course of therapy.

By leveraging non-invasive urine sampling, LAM overcomes limitations of sputum-based methods, extending diagnostic and monitoring access to pediatric, paucibacillary, and extrapulmonary TB cases. Future validation through multicenter cohorts and correlation with long-term clinical endpoints will solidify its role in global TB control, advancing the WHO vision of biomarker-guided therapeutic optimization.

## Data Availability

The raw data supporting the conclusions of this article will be made available by the authors, without undue reservation.
