# Supplementary material for: Value of urinary lipoarabinomannan levels for tuberculosis diagnosis and monitoring of therapy
Source: Front Microbiol. 2025 Aug 20;16:1653031. doi: 10.3389/fmicb.2025.1653031 (PMC12405177; doi:10.3389/fmicb.2025.1653031)
Supplement: Supplementary file 1 [file Data_Sheet_1.pdf]

Supplemental table 1. Diagnostic Performance of Different Methods for Tuberculosis

Methods		Bacteriologically Confirmed PTB		Sensitivity [95%CI]	*p* value (sensitivity)	Specificity [95%CI]	PPV [95%CI]	NPV [95%CI]	Kappa value
		positive	negative						
AFB	positive	36	0	63.16% [49.30%, 75.24%]	0.155	100.00% [93.77%, 100%]	100.00% [87.99%,100%]	77.66% [67.67%, 85.34%]	0.658
	negative	21	73						
MGIT	positive	47	0	82.46% [69.64%, 90.83%]	0.358	100.00% [93.77%, 100%]	100.00% [90.59%, 100%]	87.95% [78.51%, 93.76%]	0.841
	negative	10	73						
GeneXpert	positive	49	0	85.96% [73.65%, 93.32%]	0.154	100.00% [93.77%, 100%]	100.00% [90.94%, 100%]	90.12% [80.96%, 95.33%]	0.873
	negative	8	73						
AIMLAM	positive	43	6	75.44% [61.96%, 85.47%]	-	91.78% [82.35%, 96.61%]	87.76% [74.54%, 94.92%]	82.72% [72.36%, 89.90%]	0.683
	negative	14	67						

PPV: Positive predictive value; NPV: Negative predictive value; AFB: Acid-fast bacilli; MGIT: Mycobacteria Growth Indicator Tube.
